# Association of cardiovascular magnetic resonance diastolic indices with arrhythmia in repaired Tetralogy of Fallot

**DOI:** 10.1186/s12968-023-00928-x

**Published:** 2023-03-13

**Authors:** Sandra D. Kikano, Angela Weingarten, Sudeep D. Sunthankar, William McEachern, Kristen George-Durett, David A. Parra, Jonathan H. Soslow, Joshua D. Chew

**Affiliations:** Thomas P. Graham Division of Pediatric Cardiology Monroe Carell Jr Children’s Hospital at Vanderbilt University, 2200 Children’s Way Suite 5230, Doctors’ Office Tower, Nashville, TN 37232-9119 USA

**Keywords:** Arrhythmias, Magnetic resonance imaging, Diastolic indices, Tetralogy of Fallot

## Abstract

**Background:**

Patients with repaired Tetralogy of Fallot (rTOF) experience a high burden of long-term morbidity, particularly arrhythmias. Cardiovascular magnetic resonance (CMR) is routinely used to assess ventricular characteristics but the relationship between CMR diastolic function and arrhythmia has not been evaluated. We hypothesized in rTOF, left ventricular (LV) diastolic dysfunction on CMR would correlate with arrhythmias and mortality.

**Methods:**

Adolescents and adults with rTOF who underwent CMR were compared to healthy controls (n = 58). Standard ventricular parameters were assessed and manual planimetry was performed to generate filling curves and indices of diastolic function. Chart review was performed to collect outcomes. Univariate and multivariable logistic regression was performed to identify outcome associations.

**Results:**

One-hundred sixty-seven subjects with rTOF (mean age 32 years) and 58 healthy control subjects underwent CMR. Patients with rTOF had decreased LV volumes and increased right ventricular (RV) volumes, lower RV ejection fraction (RVEF), lower peak ejection rate (PER), peak filling rate (PFR) and PFR indexed to end-diastolic volume (PFR/EDV) compared to healthy controls. Eighty-three subjects with rTOF had arrhythmia (63 atrial, 47 ventricular) and 11 died. Left atrial (LA) volumes, time to peak filling rate (tPFR), and PFR/EDV were associated with arrhythmia on univariate analysis. PER/EDV was associated with ventricular (Odds ratio, OR 0.43 [0.24–0.80], p = 0.007) and total arrhythmia (OR 0.56 [0.37–0.92], p = 0.021) burden. A multivariable predictive model including diastolic covariates showed improved prediction for arrhythmia compared to clinical and conventional CMR measures (area under curve (AUC) 0.749 v. 0.685 for overall arrhythmia). PFR/EDV was decreased and tPFR was increased in rTOF subjects with mortality as compared to those without mortality.

**Conclusions:**

Subjects with rTOF have abnormal LV diastolic function compared to healthy controls. Indices of LV diastolic function were associated with arrhythmia and mortality. CMR diastolic indices may be helpful in risk stratification for arrhythmia.

**Supplementary Information:**

The online version contains supplementary material available at 10.1186/s12968-023-00928-x.

## Introduction

Tetralogy of Fallot (TOF) is the most common form of cyanotic congenital heart disease [1]. Repair commonly involves right ventricular (RV) ventriculotomy and infundibulotomy, RV muscle bundle resection and patching of the RV outflow tract (RVOT), with or without sparing of the pulmonary valve [1, 2]. Over time, patients with repaired TOF (rTOF) often develop RV dilation and dysfunction as well as left ventricular (LV) systolic and diastolic dysfunction. Long-term, rTOF patients experience an increasing burden of atrial arrhythmias (AA) and ventricular arrhythmias (VA) as well as sudden cardiac death [3, 4].

Accepted risk factors for morbidity and mortality in rTOF include ventricular systolic dysfunction, severe RV dilation, elevated RV systolic pressure due to RVOT obstruction, pulmonary valve dysfunction, QRS prolongation and manifest arrhythmias [2, 5–8]. These predictors help guide timing of clinical interventions such as pulmonary valve replacement, ablation procedure, and internal cardiac defibrillator implantation. Cardiovascular magnetic resonance (CMR) is routinely used in longitudinal follow up of rTOF patients [2] with predictors of adverse clinical outcomes including ventricular systolic function and RV indexed volumes [9, 10].

Diastolic dysfunction has been studied as a marker of cardiovascular disease progression and is characterized by impaired relaxation of the myocardium, leading to increased filling pressures [11]. In rTOF, diastolic dysfunction as measured by cardiac catheterization and echocardiographic indices have demonstrated clinical outcome associations including arrhythmia and decreased exercise capacity [12–15]. Despite this, noninvasive measurements of diastolic dysfunction and their clinical implications in this population are not well understood.

CMR filling curves and left atrial (LA) volumes can be used to assess diastolic dysfunction [16–18]. LA dilation and myocardial fibrosis as markers of diastolic dysfunction have the potential for use as clinical predictors for arrhythmia in rTOF [19]. To our knowledge, the association between CMR diastolic indices and arrhythmia in rTOF patients has not been evaluated. We hypothesized that CMR diastolic variables in rTOF subjects would differ from controls and associate with atrial, ventricular, and total arrhythmia (TA) burden as well as mortality in a cohort of rTOF.

## Methods

### Study population

We conducted a single-center retrospective investigation of patients evaluated at Vanderbilt University Medical Center, Nashville, Tennesee, USA. The study was approved by the Vanderbilt University Institutional Review Board. The electronic medical record was searched using an online tool developed at Vanderbilt that allows researchers to customize searches for ICD-9, ICD-10, CPT codes, and keywords. Our search for patient identification included ICD10 Q21.3, ICD9 745.2, and CPT codes associated with CMR (75,552–75,558, 75,560, and 75,565). The inclusion criteria were adults > 18 years with rTOF with pulmonary stenosis (PS), pulmonary atresia (PA), or absent pulmonary valve who had undergone at least one CMR after the age of 15 years and had at least one clinical encounter with a cardiologist. No gender-based differences were present. Individual medical charts were then reviewed by the research team. Study data were collected and managed using REDCap (Research Electronic Data Capture), an electronic data capture tool hosted at Vanderbilt University Medical Center [20]. The study cohort consisted of 167 patients with rTOF and were compared to healthy controls over 15 years old from a cohort of healthy subjects who had previously consented for non-contrasted CMR imaging as part of a separate project. The study was approved by the institutional review board with a waiver of consent for retrospective enrollment.

### Clinical data and outcomes

Chart review was performed to collect demographic and clinical data, including date of birth, gender, height, weight, age at time of CMR, original cardiac anatomy, date and age of each surgical procedure, type of surgical procedures, arrhythmia outcomes and mortality. The QRS duration was reviewed from the closest available electrocardiogram (ECG) from the time of CMR. Original anatomy was classified as TOF with PS, TOF with PA, TOF with PA and major aortopulmonary collaterals (MAPCAs), TOF with atrioventricular septal defect (AVSD) or TOF with absent pulmonary valve. Types of repairs documented were transannular patch, transannular patch with monocuspid valve, valve sparing, or RV to pulmonary artery conduit. Chart review was used to identify patients with a history of AA and VA, as well as the age of the patient at the occurrence of each arrhythmia. AA was defined as supraventricular tachycardia, atrial fibrillation, atrial flutter, and atrial tachycardia. VA was defined as non-sustained or sustained ventricular tachycardia, aborted sudden cardiac death, and sudden cardiac death. Non-sustained VA was defined as greater than 3 consecutive beats on cardiac monitoring. Sustained VA was defined as greater than 30 s. Only spontaneous arrhythmias were included.

### Cardiovascular magnetic resonance methods

#### Image acquisition

Images were obtained on a 1.5T CMR system (Avanto or Avanto Fit, Siemens Healthineers, Erlangen, Germany or Intera, Philips Healthcare, Best, The Netherlands). A balanced steady-state free precession pulse sequence (bSSFP) was used to obtain retrospectively gated cine images in the apical two chamber, left ventricular outflow tract (LVOT), apical four chamber, and short axis (SAx) views. SAx slices were obtained covering the LV from base to apex (8 mm thickness, 0 mm gap). Typical scanning parameters were: TR = 36.53 ms, TE = 1.18 ms, flip angle 80°, voxel size 1.5 × 1.5 × 8 mm, 25 phases.

#### Baseline characteristics and ventricular volumes

The first CMR performed at Vanderbilt after age 15 years was used for the study. Baseline characteristics, including date of the study, height, weight, heart rate, and blood pressure from the time of the study, and conventional ventricular parameters, including right and left ventricular end-diastolic (EDV) and end-systolic volumes (ESV), were collected from the study report. Ventricular volumes were measured by manual planimetry of the endocardium, with inclusion of the papillary muscles, in a SAx cine stack from the base of the heart to the apex. These parameters were measured by pediatric cardiologists (D.A.P., J.H.S.) experienced in CMR as part of clinical care or during enrollment of control subjects. Analysis was performed with the Leonardo Workstation (Siemens Healthineers) or the Extended MR WorkSpace (Philips Healthcare).

#### Left atrial function

**Fig. 1:**
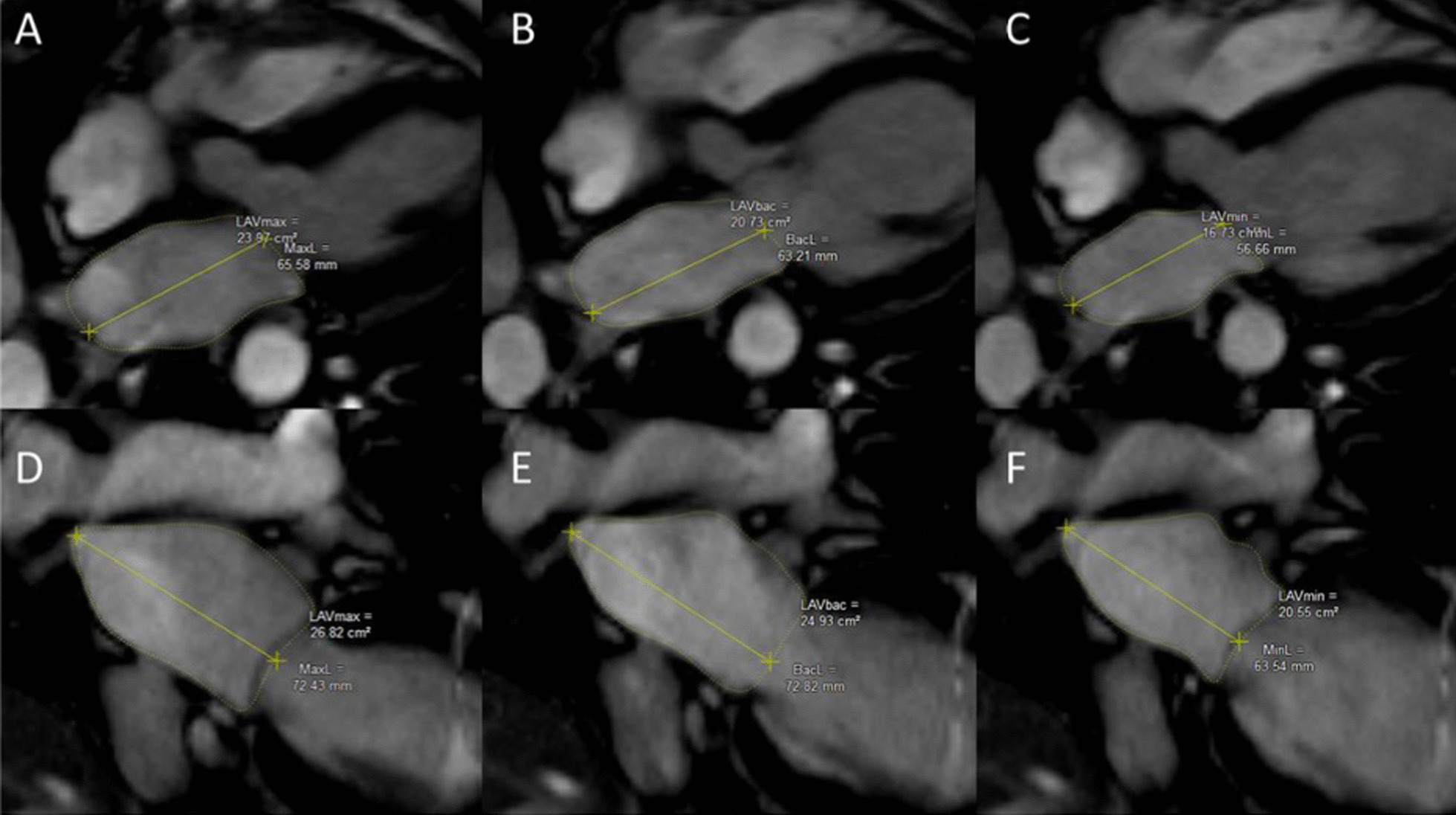
Example measurements for left atrial (LA) volume calculations for LA maximum (**A**, **D**), before atrial contraction (**B**, **E**), and minimum (**C**, **F**) volumes. *LAVmax* Left atrial maximum volume, *MaxL* Maximal length (of left atrium), *LAVBac* Left atrial volume before atrial contraction, *BacL* Maximal length before atrial contraction (of left atrium), *LAVmin* Left atrial minimum volume, *MinL* Minimum length (of left atrium)

LA volume and function were calculated as previously described [21, 22] using 4- and 2- chamber cine images by an image analyst (KGD) using Medis QMass (MedisSuite 2.1, Medis, Leiden, The Netherlands). Endocardial contours of the LA and LA length from mitral valve annulus to posterior wall of the LA were measured at maximum volume (LAV_max_), minimum volume (LAV_min_), and at pre-atrial contraction (LAV_PreA_) (Fig. 1). The time of LAV_max_ was defined as the last image before mitral valve opening. The time of LAV_min_ was defined as the first image after the closure of the mitral valve. LAV_PreA_ was determined by visual inspection as the last image before atrial contraction. LA volumes were then extrapolated from volume curves using the area-length method [22]: volume = (0.848 × area_4chamber_ × area_2chamber_)/([length_2chamber_ + length_4chamber_]/2).

To calculate LA function throughout the cardiac cycle, the following equations were used:

- Passive LA function: (LAV_max_ − LAV_PreA_)/LAV_max._
- Active LA function: (LAV_PreA_ − LAV_min_)/LAV_PreA._
- Total LA function: (LAV_max_ − LAV_min_)/LAV_max._

#### LV diastology

**Fig. 2:**
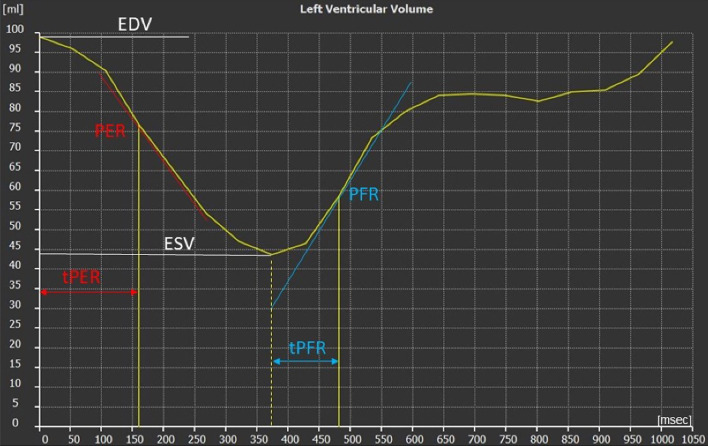
Left ventricular volume curve with diastology calculations.  EDV = end-diastolic volume; ESV = end-systolic volume; PER = peak ejection rate; PFR = peak filling rate; tPER = time to peak ejection rate; tPFR = time to peak filling rate

Manual planimetry of each phase of the cardiac cycle was performed to generate filling curves by an image analyst (KGD) with review of a subset of images by a cardiologist with over 10 years of CMR reading experience (JHS). Basilar slices without myocardium present throughout the cardiac cycle and apical slices with poor endocardial delineation were removed to improve reproducibility as per our labs protocol for filling curves [23]. Indices of diastolic function, including peak filling rate (PFR), time to peak filling (tPFR), PFR indexed to EDV (PFR/EDV), peak ejection rate (PER), time to peak ejection (tPER), and PER to EDV (PER/EDV), were automatically generated by QMass [16, 23] (Fig. 2). After generating a LV time-volume curve with instantaneous filling rates plotted over time, diastolic indices were defined as the following:

1. PFR: maximal increase in LV volume over time, which correlates to the maximal positive slope in the volume curve occurring in early diastole
2. tPFR: time interval from the end-systole phase to PFR
3. PER: maximal decrease in LV volume over time, which correlates to the maximal negative slope in the volume curve occurring in systole
4. tPER: time interval between end-diastolic phase to PER
5. Diastolic dysfunction is characterized by decreased PFR as well as prolonged tPFR.

### Statistical analysis

Patient characteristics and CMR data associations were compared using Pearson’s Chi-squared for categorical variables and Wilcoxon rank sum for continuous variables. Data is presented as bootstrapped means with 1000 repetitions (95% confidence interval [CI] of the mean). Univariate logistic regression was performed to identify CMR and clinical predictors associated with arrhythmia. Odds ratios are reported with 95% CIs. Stepwise forward selection was then used to fit a multivariable model that maximized outcome prediction with factors considered to be significant at the 0.05 level. Receiver-operating characteristic (ROC) curves were generated with a calculated Area Under the Curve (AUC) to quantify arrhythmia prediction. For time to event analysis, patients with arrhythmia following CMR were analyzed with Cox proportional hazard regression models to estimate associations of each predictor with time of onset of arrythmia. Survival analyses were performed for VA using LV volumes, and LV diastolic variables (PER and tPER). Survival curves were compared using the log-rank test. Due to small sample size of patient developing arrhythmia following CMR analysis in this study, the time-to -event analysis was felt to be underpowered and is included as a supplement to the main manuscript. A linear regression analysis was also completed correlating pulmonary regurgitation and QRS duration with diastolic predictors to analyze if factors affecting ventricular septal position may affect diastolic variables. A commercially available statistical software package was used for data analysis (STATA, version 17.0, College Station, Texas, USA).

## Results

### Patient demographics

One hundred sixty-seven rTOF and 58 healthy control subjects were included. Table 1 summarizes baseline characteristics rTOF and healthy control group subjects at the time of CMR. The bootstrapped mean age of rTOF patients was 32.4 years (95% CI [30.4–34.4]) and 50.3% were male. The original anatomy of rTOF patients included 81.4% categorized as TOF with PS, 13.8% with PA, 2.4% with PA with MAPCAs, 1.8% with absent pulmonary valve, and 0.6% with TOF with AVSD. Most patients (75%) underwent rTOF with trans-annular patch, 12% underwent valve-sparing repair, and 13% were repaired with RV-Pulmonary Artery conduit. The mean age of repair was 59 months [47–71]. At the time of analysis 49% had undergone at least one pulmonary valve replacement (PVR) at a mean age of 26.8 years [26.8–32.4]. rTOF patients with ECG near the time of CMR (N = 162) also had a mean QRS duration of 141 ms [137–147].

**Table 1.** Demographic characteristics of control vs. rTOF patients

	Healthy Controls (N = 58)	rTOF (N = 167)	P value
Male	41 (70.7%)	84 (50.3%)	0.007
Age (years)	28.1 (25.5–30.7)	32.4 (30.4–34.4)	0.038
Height (cm)	172.7 (168.1–177.3)	165.6 (163.3–167.9)	< 0.001
Weight (kg)	73.8 (70.2–77.4)	74.9 (71.2–78.5)	0.767
BSA (m^2^)	1.88 (1.82–1.94)	1.85 (1.81–1.90)	0.373
Heart rate (bpm)	68 (65–72)	77 (75–79)	< 0.001
QRS Duration (msec)		141 (137–147)	

*BSA* body surface area, *rTOF* tetralogy of Fallot

*Data presented as: bootstrapped mean with 1000 repetitions (95% confidence interval of the mean)

*P-values: Wilcoxon rank sum for continuous variables, χ2 test for categorical variables

### CMR parameters in rTOF versus control

Conventional volumes, LA parameters and LV diastolic data of rTOF patients versus control subjects are shown in Table 2. Compared to controls, rTOF patients had lower LV volumes (mean LVEDV index (LVEDVI) 67.2 ml/m^2^ [64.1–70.3] v. 84.6 [81.0–88.0], p < 0.001), larger RV volumes (mean RVEDV index (RVEDVI) 123.8 ml/m^2^ [116.6–131.0] v. 86.3 [82.3–90.4], p < 0.001), and lower RV ejection fraction (RVEF) (48.1% [46.5–49.7] v. 57.6% [55.8–59.3], p < 0.001). LV ejection fraction (LVEF) did not differ (p = 0.997). Total and passive LA function were lower in rTOF patients (51.1% [59.8–63.4], p < 0.001 v. 61.7% [59.8–63.4], p < 0.001 for total), (26.5% [24.7–28.3], p < 0.001 v. 39.5% [37.2–41.8], p < 0.001 for passive). Active LA function did not significantly differ (p = 0.074). LV diastolic indices showed that rTOF subjects had lower PER (311 ml/s [297–325] v. 386 [364–409], p < 0.001), PFR (297 ml/s [281–314] v. 427 [403–451], p < 0.001) and PFR/EDV (2.69 s^−1^ [2.57–2.80] v. 3.18 [3.03–3.33] p < 0.001) compared to healthy controls.

**Table 2:** CMR characteristics of control vs. rTOF patients

	Healthy Controls (N = 58)	rTOF (N = 167)	P value
CMR conventional ventricular parameters			
LVEDVI (ml/m^2^)	84.6 (81.0–88.2)	67.2 (64.1–70.3)	< 0.001
LVESVI (ml/m^2^)	33.3 (31.5–35.1)	27.7 (25.6–29.8)	< 0.001
LVEF (%)	60.8 (59.7–62.0)	59.9 (58.2–61.5)	0.997
RVEDVI (ml/m^2^)	86.3 (82.3–90.4)	123.8 (116.6–131.0)	< 0.001
RVESVI (ml/m^2^)	36.5 (34.5–38.4)	66.1 (61.1–71.0)	< 0.001
RVEF (%)	57.6 (55.8–59.3)	48.1 (46.5–49.7)	< 0.001
LA volume and function vs. controls			
Indexed LA_max_ Vol. (ml/m^2^)	36.1 (33.8–38.4)	30.7 (28.6–32.8)	< 0.001
Indexed LA_min_ Vol. (ml/m^2^)	13.9 (12.7–15.1)	15.4 (13.8–16.9)	0.715
Indexed LA PAC Vol. (ml/m^2^)	21.9 (20.1–23.8)	22.8 (21.0–24.6)	0.745
Total LA Function (%)	61.7 (59.8–63.4)	51.1 (49.3–53.0)	< 0.001
Passive LA Function (%)	39.5 (37.2–41.8)	26.5 (24.7–28.3)	< 0.001
Active LA Function (%)	65.9 (64.6–67.2)	63.1 (61.7–64.5)	0.0736
LV diastology			
PER (ml/s)	386 (364–409)	311 (297–325)	< 0.001
tPER (ms)	145 (139–152)	137 (131–144)	0.174
PER/EDV (s^−1^)	2.84 (2.75–2.95)	3.65 (2.05–5.26)	0.417
PFR (ml/s)	427 (403–451)	297 (281–314)	< 0.001
tPFR (ms)	140 (134–146)	146 (138–154)	0.778
PFR/EDV (s^−1^)	3.18 (3.03–3.33)	2.69 (2.57–2.80)	< 0.001

*rTOF* tetralogy of Fallot, *LVEDVI* left ventricular end-diastolic volume index, *LVESVI* left ventricular end-systolic volume index, *LVEF* left ventricular ejection fraction, *RVEDVI* right ventricular end-diastolic volume index, *RVESVI* right ventricular end-systolic volume index, *RVEF* right ventricular ejection fraction, *LA* left atrium, *PER* peak ejection rate, *tPER* time to peak ejection rate, *PER/EDV* peak ejection rate to end diastolic volume, *PFR* peak filling rate, *tPFR* time to peak filling rate, *PFR/EDV* peak filling rate to end diastolic volume

*Data presented as: bootstrapped mean with 1000 repetitions (95% confidence interval of the mean)

*P-values: Wilcoxon rank sum

### Arrhythmia and clinical outcomes

Eighty-three rTOF subjects had an arrhythmia, with 63 experiencing an AA and 47 experiencing VA. Of these, 42 (51%) had arrhythmia present prior to date of CMR, while 41 (49%) developed arrhythmia following CMR and one developed arrhythmia on the date of CMR. The mean time of arrhythmia diagnosis prior to CMR was 5.3 years [95% 3.0–7.6]. Older age at the time of repair was associated with increased AA (OR 1.01 per month, p = 0.005) and TA (OR 1.01 per month, p = 0.010) (Table 3). When analyzing conventional CMR indices, increased RVEDVI was associated with increased AA (OR 1.08 per 10 mL/m^2^, p = 0.032) and VA (OR 1.10 per 10 mL/m^2^, p = 0.029). Increased RVESV index (RVESVI) was associated with increased AA (1.16 per 10 mL/m^2^, p = 0.009), VA (1.18 per 10 mL/m^2^, p = 0.010), and TA (OR 1.14 per 10 mL/m^2^, p = 0.016). Increased RVEF was associated with decreased AA (OR 0.95, p = 0.005), VA (OR 0.95, p = 0.007), TA (OR 0.96, p = 0.009), while LVEF did not have significant association with arrhythmia (p = 0.673 for TA) (Table 3).

**Table 3:** Arrhythmia outcomes

	OR AA	P value	OR VA	P value	OR TA	P value
Age (years)	1.09 (1.06–1.13)	< 0.001	1.06 (1.02–1.09)	< 0.001	1.08 (1.05–1.11)	< 0.001
QRS	1.03 (1.01–1.04)	< 0.001	1.02 (1.01–1.03)	0.005	1.02 (1.01–1.03)	< 0.001
Age at repair (months)	1.01 (1.00–1.01)	0.005	1.00 (1.00–1.01)	0.113	1.01 (1.00–1.01)	0.010
LVEDVI ( per 10 ml/m^2^)	1.13 (0.94–1.36)	0.181	1.34 (1.09–1.64)	0.005	1.15 (0.98–1.35)	0.088
LVESVI (per 10 ml/m^2^)	1.16 (0.89–1.51)	0.278	1.43 (1.07–1.91)	0.015	1.20 (0.95–1.52)	0.122
LVEF (%)	1.00 (0.97–1.03)	0.989	0.98 (0.95–1.02)	0.311	0.99 (0.97–1.02)	0.673
RVEDVI (per 10 ml/m^2^)	1.08 (1.01–1.17)	0.032	1.10 (1.01–1.19)	0.029	1.07 (1.00–1.15)	0.056
RVESVI (per 10 ml/m^2^)	1.16 (1.04–1.29)	0.009	1.18 (1.04–1.33)	0.010	1.14 (1.02–1.26)	0.016
RVEF (%)	0.95 (0.92–0.98)	0.005	0.95 (0.92–0.99)	0.007	0.96 (0.93–0.99)	0.009
Indexed LA_max_ Vol. (per 10 ml/m^2^)	2.03 (1.36–3.02)	< 0.001	1.64 (1.11–2.44)	0.014	1.74 (1.22–2.46)	0.002
Indexed LA_min_ Vol. (per 10 ml/m^2^)	2.85 (1.53–5.30)	0.001	2.30 (1.22–4.32)	0.010	2.31 (1.33–4.02)	0.003
Indexed LA BAC Vol. (ml/m^2^)	1.09 (1.04–1.14)	0.001	1.06 (1.01–1.11)	0.011	1.06 (1.02–1.11)	0.003
Total LA Fxn (%)	0.97 (0.94–1.00)	0.046	0.97 (0.94–1.00)	0.067	0.98 (0.95–1.00)	0.094
Passive LA Fxn (%)	0.98 (0.94–1.01)	0.159	0.97 (0.94–1.00)	0.067	0.98 (0.95–1.00)	0.094
Active LA Fxn (%)	1.01 (0.97–1.04)	0.759	0.99 (0.95–1.04)	0.793	1.01 (0.97–1.04)	0.712
PER (per 10 ml/s)	1.03 (0.99–1.06)	0.127	1.02 (0.99–1.06)	0.230	1.02 (0.98–1.05)	0.280
tPER (per 10 ms)	1.05 (0.97–1.13)	0.250	1.06 (0.97–1.15)	0.187	1.06 (0.99–1.15)	0.093
PER/EDV (s^−1^)	0.64 (0.39–1.05)	0.076	0.43 (0.24–0.8)	0.007	0.585 (0.37–0.92)	0.021
PFR (per 10 ml/s)	1.00 (0.97–1.03)	0.990	1.01 (0.97–1.04)	0.711	1.00 (0.97–1.03)	0.954
tPFR (per 10 ms)	1.13 (1.04–1.22)	0.003	1.12 (1.03–1.22)	0.007	1.10 (1.03–1.18)	0.007
PFR/EDV (s^−1^)	0.51 (0.32–0.82)	0.005	0.44 (0.24–0.75)	0.003	0.56 (0.37–0.85)	0.006

*LVEDVI* left ventricular end-diastolic volume index, *LVESVI* left ventricular end-systolic volume index, *LVEF* left ventricular ejection fraction, *RVEDVI* right ventricular end-diastolic volume index, *RVESVI* right ventricular end-systolic volume index, *RVEF* right ventricular ejection fraction, *LA* left atrium, *PER* peak ejection rate, *tPER* time to peak ejection rate, *PER/EDV* peak ejection rate to end diastolic volume, *PFR* peak filling rate; *tPFR* time to peak filling rate, *PFR/EDV* peak filling rate to end diastolic volume

*Data presented as odds ratios (OR) with 95% CIs for logistic regression for each clinical or imaging variable associated with outcome of arrhythmia

Analysis of diastolic indices showed all LA volume measurements were positively associated AA, VA, and TA (Table 3). The most significant associated measure in this group was indexed LA minimum volumes, with OR between 2.30–2.85 per 10 mL/m^2^ for each subtype of arrhythmia. Total LA function was associated with AA (OR 0.97, p = 0.046). The remainder of LA function measurements were not significantly related to arrhythmia. LV diastolic indices showed that tPFR (OR 1.10 per 10 ms, p = 0.007 for TA) and PFR/EDV (OR 0.56, p = 0.006 for TA) were associated with all types arrhythmia, with worsening of diastolic dysfunction conferring increased association of arrhythmia (Table 3).

In patients who had contrast CMR performed, LGE (not including the RV insertion points) was also assessed. Forty-two patients underwent contrast CMR and 15 (36%) of these subjects had LGE. On univariate analysis, the presence of LGE was not associated with AA (OR 1.14, p = 0.874), VA (OR 1.90, p = 0.388), or TA (OR 1.19, p = 0.804).

There were 11 subjects with known mortality in the rTOF cohort. PFR/EDV was decreased and tPFR was increased in rTOF subjects with mortality as compared to those without mortality (PFR/EDV 1.94 s^−1^ [1.57–2.30] v. 2.74 [2.62–2.86], p < 0.001), (tPFR 207 ms [143–271] v. 141 [134–148], p = 0.020) (Table 4). Given the small sample size of patients, multivariable analysis was not performed for mortality.

**Table 4:** Mortality Outcomes

	No Mortality (N = 154)	Mortality (N = 11)	P value
Male	76 (49.4%)	6 (54.5%)	
Age (years)	31.2 (29.3–33.0)	48.7 (43.0–54.4)	< 0.001
LVEDVI (ml/m^2^)	66.1 (62.8–69.3)	82.0 (71.7–92.3)	0.003
LVESVI (ml/m^2^)	26.6 (24.4–28.8)	40.0 (31.8–48.3)	0.001
LVEF (%)	60.7 (59.1–62.3)	51.9 (45.2–58.5)	0.009
RVEDVI (ml/m^2^)	122.7 (115.7–129.6)	142.3 (106.1–178.5)	0.371
RVESVI (ml/m^2^)	65.3 (60.2–70.4)	78.3 (46.9–109.6)	0.682
RVEF (%)	48.0 (46.3–49.7)	48.5 (42.9–54.1)	0.746
LA volume and function by mortality in rTOF (restricted to subjects with LA volume)			
Indexed LA_max_ Vol. (ml/m^2^)	29.5 (27.6–31.4)	45.9 (35.0–56.7)	< 0.001
Indexed LA_min_ Vol. (ml/m^2^)	14.5 (13.0–16.0)	26.5 (19.4–33.5)	< 0.001
Indexed LA BAC Vol. (ml/m^2^)	21.7 (19.9–23.4)	37.2 (29.1–45.3)	< 0.001
Total LA Fxn (%)	51.7 (49.7–53.7)	43.4 (36.9–50.0)	0.029
Passive LA Fxn (%)	27.2 (25.4–29.1)	18.5 (13.4–23.5)	0.012
Active LA Fxn (%)	63.0 (61.5–64.4)	62.9 (56.4–69.3)	0.931
LV diastology			
PER (ml/s)	310 (295–325)	321 (259–382)	0.746
tPER (ms)	136 (130–143)	148 (121–175)	0.426
PER/EDV (s^−1^)	3.76 (2.06–5.47)	2.10 (1.93–2.28)	< 0.001
PFR (ml/s)	297 (280–314)	296 (220–373)	0.830
tPFR (ms)	141 (134–149)	207 (146–267)	0.018
PFR/EDV (s^−1^)	2.74 (2.62–2.86)	1.94 (1.58–2.30)	0.001

*LVEDVI* left ventricular end-diastolic volume index, *LVESVI* left ventricular end-systolic volume index, *LVEF* left ventricular ejection fraction, *RVEDVI* right ventricular end-diastolic volume index, *RVESVI* right ventricular end-systolic volume index, *RVEF* right ventricular ejection fraction, *LA* left atrium, *PER* peak ejection rate, *tPER* time to peak ejection rate, *PER/EDV* peak ejection rate to end diastolic volume, *PFR* peak filling rate, *tPFR* time to peak filling rate, *PFR/EDV* peak filling rate to end diastolic volume

*Data presented as: bootstrapped mean with 1000 repetitions (95% confidence interval of the mean)

*P-values: continuous Mann–Whitney U tests

Linear regression of diastolic variables with factors that may affect septal position including degree of pulmonary insufficiency and QRS duration are shown in Additional file 1: Table S1 and Additional file 2: Table S2. The analysis with pulmonary insufficiency demonstrated poor correlation with all diastolic variables. For QRS correlates, there were a few statistically significant relationships with low r-squared values suggesting unclear clinical significance.

### Multivariable analysis of arrhythmia

**Fig. 3:**
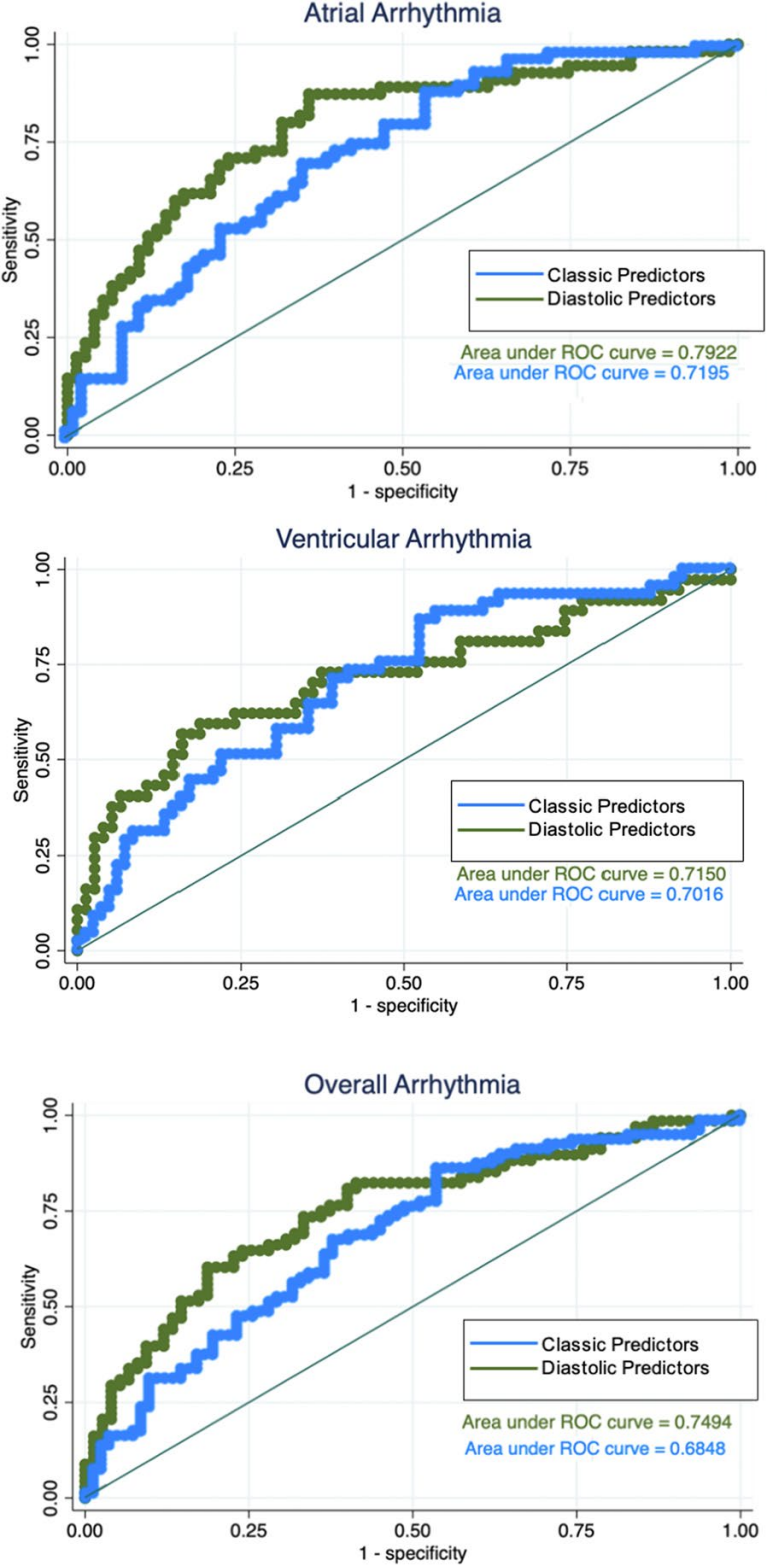
Comparing ROC Curves for Conventional CMR and Clinical Characteristics vs. Diastolic CMR and Clinical Variables

An initial multivariable model was made using conventical CMR and clinical variables that were significant in univariate analysis and that have previously been described as predictive of clinical outcomes [5, 9, 24]. These included QRS duration, RVEDVI, RVESVI, and RVEF as continuous variables for each type of arrhythmia (AA, VA, and TA). QRS duration was the only variable that significantly differentiated in all arrhythmia subsets (Table 5). A subsequent multivariable analysis was then performed using diastolic markers that were significant in univariate analysis as well as QRS duration. By multivariable logistic regression analysis, the best model for predicting AA and TA included QRS duration, LA indexed minimum volume, and TPFR. For VA prediction, the best model included QRS duration, LA indexed minimum volume, and PFR/EDV. In all subset analyses, this model for arrhythmia prediction demonstrated improved AUC statistics compared to the model using conventional parameters (Fig. 3). To account for age at the time of CMR in the models given risk for diastolic dysfunction with increasing age, an additional multivariate model that includes age at the time of CMR was assessed with comparison to classic predictors (Additional file 1: Table S3) with similar results to our diastolic predictor models without age.

**Table 5:** Multivariable analysis of variables associated with arrhythmia

Atrial arrhythmia			
Model 1: Classic Predictors (area under ROC curve = 0.72)			
	OR	p-value	95% CI
QRS Duration	1.03	0.001	1.01–1.04
RVEDVI (per 10 ml/m^2^)	1.25	0.330	0.80–1.95
RVESVI (per 10 ml/m^2^)	0.72	0.398	0.33–1.55
RVEF (%)	0.95	0.306	0.86–1.05
**Model 2: Including Diastolic Predictors (area under ROC curve = 0.79)**			
QRS duration	1.02	0.010	1.00–1.03
Indexed LA_Min_ Vol. (per 10 ml/m^2^)	3.20	0.004	1.46–7.04
tPFR	1.01	0.065	1.00–1.02

Ventricular arrhythmia			
Model 1: Classic Predictors (area under ROC curve = 0.70)			
	OR	p-value	95% CI
QRS Duration	1.01	0.060	1.00–1.03
RVEDVI (per 10 ml/m^2^)	1.42	0.156	0.88–2.30
RVESVI (per 10 ml/m^2^)	0.59	0.210	0.26–1.35
RVEF (%)	0.91	0.098	0.81–1.02
**Model 2: Including Diastolic Predictors (area under ROC curve = 0.72)**			
QRS duration	1.01	0.160	1.00–1.03
Indexed LA_min_ Vol. (per 10 ml/m^2^)	2.19	0.035	1.05–4.53
PFR/EDV	0.61	0.127	0.32–1.15

Total arrhythmia			
Model 1: Classic Predictors (area under ROC curve = 0.68)			
	OR	p-value	95% CI
QRS Duration	1.02	0.003	1.01–1.03
RVEDVI (per 10 ml/m^2^)	1.16	0.467	0.78–1.74
RVESVI (per 10 ml/m^2^)	0.80	0.535	0.40–1.62
RVEF (%)	0.96	0.362	0.88–1.05
**Model 2: Including Diastolic Predictors (area under ROC curve = 0.75)**			
QRS duration	1.02	0.004	1.01–1.03
Indexed LA_min_ Vol. (per 10 ml/m^2^)	2.35	0.010	1.22–4.51
tPFR	1.01	0.146	1.00–1.01

RVEDVI- right ventricular end-diastolic volume index; RVESVI- right ventricular end-systolic volume index; RVEF- right ventricular ejection fraction; LA- left atrium; PER- peak ejection rate; tPER- time to peak ejection rate; PER/EDV- peak ejection rate to end diastolic volume; PFR- peak filling rate; tPFR- time to peak filling rate; PFR/EDV- peak filling rate to end diastolic volume

^*^Data presented as odds ratios (OR) with 95% CI for multivariable logistic regression

### Time to event analysis

In patients with arrhythmia following CMR, there were 28 AA and 24 VA. The mean follow-up period from initial CMR was 7.5 years [6.9–8.2]. Time to arrhythmia analysis was limited due to small sample size of patients with arrythmia following CMR and was felt to be underpowered. Cox proportional hazard ratios to assess risk of onset of all arrhythmias with conventional, LA, and LV diastolic CMR parameters are shown in Additional file 1: Table S4. For time to new AA, there was an associated risk with increased PER (HR 1.005, p = 0.032). LVEDVI (HR 1.014, p = 0.013), LVESV index (LVESVI) (HR 1.017, p = 0.022), PER (HR 1.005, p = 0.022), and tPER (HR 1.021,p = 0.001) were associated with new onset of VA. Overall new arrhythmia risk was associated with tPER (HR 1.012, p = 0.007).

## Discussion

Our data suggest that adolescent and adult rTOF subjects have abnormal LV diastolic function compared to healthy controls. Previous studies have demonstrated important echocardiographic, ECG, clinical, and CMR factors that are predictors of worse clinical outcomes with a focus on ventricular size, systolic dysfunction, and pulmonary insufficiency. Other described clinical predictors include QRS duration and vector magnitude [8, 25] as a proxy for conduction delay in the setting of ventricular scarring and dysfunction. Severely depressed LV systolic function [8, 26] has been associated with sudden cardiac death, however the search for earlier clinical predictors has been challenging and improving models for risk prediction continues to be an area of active research.

In comparing rTOF patients to controls, our cohort of rTOF patients had smaller LV volumes, preserved LV systolic function, larger RV volumes, and decreased RV systolic function. On comparison of diastolic metrics, rTOF patients had decreased LA_max_, decreased total and passive LA function, decreased PER, PFR, and PFR/EDV. Based on these findings, patients with rTOF had a higher incidence of diastolic dysfunction than that of the healthy control group.

Diastolic dysfunction is prevalent in rTOF and understanding its clinical importance is an active area of study. DiLorenzo et. al investigated diastolic dysfunction in rTOF as measured by echocardiography in comparison to cardiac catheterization data [27]. The investigators did not show an association between Doppler E/e’ and RV filling pressures by cardiac catheterization. Meanwhile, Aboulhosm et. al showed Doppler indices indicative of RV and LV diastolic dysfunction did correlate with increased presence of ventricular arrhythmia in patients with rTOF [12].

CMR has been the gold standard for imaging adolescents and adults with rTOF [2, 25, 28]. This study aimed to investigate the association of diastolic CMR measures with arrhythmia in rTOF. Univariate analysis showed increased prevalence of arrhythmia in rTOF patients with increased LA volumes, while LA function did not demonstrate a significant association. Worsening LV diastolic dysfunction, as assessed by tPFR and PFR/EDV, associated with presence of all types of arrhythmia. In addition, worsening systolic function, as assessed with PER/EDV, also associated with increases in ventricular and overall arrhythmia. LGE analysis was limited in this study group by frequency of non-contrasted CMR performed.

Multivariable analysis suggests that CMR diastolic predictors have a stronger correlation with all arrhythmia outcomes in our cohort. The classical models used for comparison were chosen based on the 2018 American Heart Association (AHA) guidelines for adult congenital patients [2] including RVEDVI and RVESVI as well as decreased systolic function.

Time to event analysis for arrhythmia was limited due to the limited number of patients with arrhythmia following CMR in this retrospective study. Conventional and diastolic parameters related to LV volumes and diastolic function were associated with arrhythmia, although these results are likely underpowered.

The etiology of diastolic dysfunction is likely multifactorial and possibly related to history of cardiopulmonary bypass and chronic changes in rTOF. Previous studies have shown the relationship of LV dysfunction and adverse clinical outcomes, highlighting the importance of ventricular-ventricular interactions in this population [28, 29]. Impaired ventricular relaxation and compliance may also lead to LA changes and subsequent LA remodeling, which has a strong correlation with pathologic arrhythmias [11, 22, 30]. Right atrial volumes, although not measured in this study likely also have a role in arrhythmia, especially atrial foci for arrhythmia which has been previously shown in this population, especially in patients with LV dysfunction [31]. Chronic pulmonary insufficiency as well as other right sided valvular changes, RV scarring, and ventricular septal defect patches also lead to abnormal RV wall motion, increased RV filling pressures, and decreased RV compliance. This study also suggests that indices of LV diastolic function associate with arrhythmias and mortality in adolescent and adult rTOF subjects. CMR diastolic indices may help in risk stratification for arrhythmia morbidity in rTOF and demonstrate the importance of ventricular interactions in this population.

The ability to risk stratify rTOF patients who are at risk for arrhythmia and sudden cardiac death continues to be an important area of study. Diastolic indices may be an important clinical predictor of arrhythmia in patients with rTOF. Future analyses should prospectively investigate the additive benefit of diastolic indices to current risk stratification strategies in rTOF.

## Limitations

This study was retrospective in nature and was underpowered in the analysis of time course for outcomes. Additionally, this study was performed at a single center which may represent potential selection bias. CMR and arrhythmia analysis was conducted at one point in time and may not reflect dynamic changes in outcomes. The improvements in arrhythmia prediction modeling in this cohort with the addition of diastolic measures is coupled with the increased provider burden to perform these on CMR analysis. These measures are not routinely automated and it is unclear if the benefits in clinical outcomes in this retrospective study would lead to improved prospective clinical outcomes. With improving automated features in CMR analysis software, implementation of automated algorithms show promise with validation in automating CMR measures including those of diastolic function [32]. Additionally, RV diastolic dysfunction was not measured in this study due to the complexity of RV geometry, filling, and tricuspid valve inflow. A recent meta-analysis demonstrated that other markers of RV diastolic dysfunction, including end-diastolic forward flow, may not be specific for restrictive RV physiology, demonstrating the complexity associated with CMR assessment of RV diastolic dysfunction [34]. Some studies suggest that more accurate assessments of RV diastology may be achieved with four-dimensional CMR [33]. Future studies should evaluate the association of RV diastolic CMR measures with arrhythmia and mortality.

## Conclusions

CMR diastolic indices are associated with arrhythmias in adolescent and adult rTOF patients. These results suggest that CMR indices may be helpful in risk stratification for arrhythmia morbidity. Future analyses should prospectively investigate the additive benefit of assessment of diastolic indices to current rTOF risk stratification strategies.

## Supplementary Information

**Additional file 1: Table S1. **Linear regression for all diastolic variables correlation with main pulmonary artery regurgitant fraction.

**Additional file 2:** **Table S2. **Linear regression for all diastolic variables correlation with main pulmonary artery regurgitation fraction and QRS duration.

**Additional file 3: Table S3. **Multivariable Analysis of Variables Associated with Arrhythmia including age at CMR analysis.

**Additional file 4: Table S4. **Time to event analysis for arrhythmia.

## Data Availability

The datasets used and/or analyzed during the current study are available from the corresponding author on reasonable request.

## Abbreviations

AA: Atrial arrhythmia
AHA: American Heart Association
AUC: Area under the curve
AVSD: Atrioventricular septal defect
bSSFP: Balanced steady state free precession
CMR: Cardiovascular magnetic resonance
ECG: Electrocardiogram
EDV: End diastolic volume
ESV: End-systolic volumes
LA: Left atrium
LAV_max_: Left atrial maximum volume
LAV_min_: Left atrial minimum volume
LAV_PreA_: Pre atrial contraction left atrial volume
LV: Left ventricle/left ventricular
LVEDVI: Left ventricular end-diastolic volume index
LVEF: Left ventricular ejection fraction
LVESVI: Left ventricular end-systolic volume index
LVOT: Left ventricular outflow tract
MAPCAs: Major aortopulmonary collaterals
PA: Pulmonary atresia
PER: Peak ejection rate
PFR: Peak filling rate
PS: Pulmonary stenosis
PVR: Pulmonic valve replacement
REDCap: Research Electronic Data Capture
ROC: Receiver-operator curve
rTOF: Repaired tetralogy of Fallot
RV: Right ventricle, right ventricular
RVEDVI: Right ventricular end-diastolic volume index
RVEF: Right ventricular ejection fraction
RVESVI: Right ventricular end-systolic volume index
RVOT: Right ventricular outflow tract
SAx: Short axis
TA: Total arrhythmia
TOF: Tetralogy of Fallot
tPER: Time to peak ejection
tPFR: Time to peak filling
VA: Ventricular arrhythmia
