# Supplementary material for: Association of cardiovascular magnetic resonance diastolic indices with arrhythmia in repaired Tetralogy of Fallot
Source: J Cardiovasc Magn Reson. 2023 Mar 13;25:17. doi: 10.1186/s12968-023-00928-x (PMC10009941; doi:10.1186/s12968-023-00928-x)
Supplement: Supplementary file 1 — Additional file 1: Table S1. Linear regression for all diastolic variables correlation with main pulmonary artery regurgitant fraction. [file 12968_2023_928_MOESM1_ESM.docx]

**Additional file 1: Table S1.** Linear regression for all diastolic variables correlation with main pulmonary artery regurgitant fraction

|  | Adjusted R-squared | p-value |
| --- | --- | --- |
| Indexed LA_max_ Vol. (per 10 ml/m^2^) | 0.007 | 0.300 |
| Indexed LA_min_ Vol. (per 10 ml/m^2^) | 0.004 | 0.223 |
| Indexed LA BAC Vol. (ml/m^2^) | 0.001 | 0.301 |
| Total LA Fxn (%) | -0.005 | 0.513 |
| Passive LA Fxn (%) | -0.008 | 0.760 |
| Active LA Fxn (%) | -0.008 | 0.935 |
| PER (per 10 ml/s) | -0.007 | 0.832 |
| tPER (per 10 ms) | 0.008 | 0.150 |
| PER/EDV (s^-1^) | 0.002 | 0.267 |
| PFR (per 10 ml/s) | -0.001 | 0.365 |
| tPFR (per 10 ms) | -0.007 | 0.807 |
| PFR/EDV (s^-1^) | -0.001 | 0.340 |
