# Supplementary material for: Association of cardiovascular magnetic resonance diastolic indices with arrhythmia in repaired Tetralogy of Fallot
Source: J Cardiovasc Magn Reson. 2023 Mar 13;25:17. doi: 10.1186/s12968-023-00928-x (PMC10009941; doi:10.1186/s12968-023-00928-x)
Supplement: Supplementary file 2 — Additional file 2: Table S2. Linear regression for all diastolic variables correlation with main pulmonary artery regurgitation fraction and QRS duration. [file 12968_2023_928_MOESM2_ESM.docx]

**Additional file 2: Table S2.** Linear regression for all diastolic variables correlation with main pulmonary artery regurgitation fraction and QRS duration

|  | Adjusted R-squared | p-value |
| --- | --- | --- |
| Indexed LA Max Vol. (per 10 ml/m^2^) | -0.003 | 0.470 |
| Indexed LA Min Vol. (per 10 ml/m^2^) | 0.027 | 0.027 |
| Indexed LA BAC Vol. (ml/m^2^) | 0.013 | 0.094 |
| Total LA Fxn (%) | 0.111 | <0.001 |
| Passive LA Fxn (%) | 0.065 | 0.001 |
| Active LA Fxn (%) | -0.007 | 0.946 |
| PER (per 10 ml/s) | 0.039 | 0.007 |
| tPER (per 10 ms) | -0.002 | 0.004 |
| PER/EDV (s^-1^) | 0.013 | 0.084 |
| PFR (per 10 ml/s) | -0.001 | 0.960 |
| tPFR (per 10 ms) | 0.014 | 0.074 |
| PFR/EDV (s^-1^) | 0.076 | <0.001 |
