# Supplementary material for: Association of cardiovascular magnetic resonance diastolic indices with arrhythmia in repaired Tetralogy of Fallot
Source: J Cardiovasc Magn Reson. 2023 Mar 13;25:17. doi: 10.1186/s12968-023-00928-x (PMC10009941; doi:10.1186/s12968-023-00928-x)
Supplement: Supplementary file 3 — Additional file 3: Table S3. Multivariable Analysis of Variables Associated with Arrhythmia including age at CMR analysis. [file 12968_2023_928_MOESM3_ESM.docx]

**Additional file 3: Table S3.** Multivariable Analysis of Variables Associated with Arrhythmia including age at CMR analysis

| **Atrial Arrhythmia** | | | |
| --- | --- | --- | --- |
| Model 1: Classic Predictors (area under ROC curve = 0.72) | | | |
|  | OR | p-value | 95% CI |
| QRS Duration | 1.03 | 0.001 | 1.01-1.04 |
| RVEDVI (per 10 ml/m^2^) | 1.25 | 0.330 | 0.80-1.95 |
| RVESVI (per 10 ml/m^2^) | 0.72 | 0.398 | 0.33-1.55 |
| RVEF (%) | 0.95 | 0.306 | 0.86-1.05 |
| Model 2: Including Diastolic Predictors (area under ROC curve = 0.84) | | | |
| Age (years) | 1.07 | 0.001 | 1.03-1.12 |
| QRS duration | 1.02 | 0.010 | 1.00-1.04 |
| Indexed LA_min_ Vol. (per 10 ml/m^2^) | 2.19 | 0.056 | 0.98-4.91 |
| tPFR | 1.01 | 0.241 | 1.00-1.02 |
| **Ventricular Arrhythmia** | | | |
| Model 1: Classic Predictors (area under ROC curve = 0.70) | | | |
|  | OR | p-value | 95% CI |
| QRS Duration | 1.01 | 0.060 | 1.00-1.03 |
| RVEDVI (per 10 ml/m^2^) | 1.42 | 0.156 | 0.88-2.30 |
| RVESVI (per 10 ml/m^2^) | 0.59 | 0.210 | 0.26-1.35 |
| RVEF (%) | 0.91 | 0.098 | 0.81-1.02 |
| Model 2: Including Diastolic Predictors (area under ROC curve = 0.72) | | | |
| Age (years) | 1.02 | 0.296 | 0.98-1.07 |
| QRS duration | 1.01 | 0.134 | 1.00-1.03 |
| Indexed LA_min_ Vol. (per 10 ml/m^2^) | 1.93 | 0.086 | 0.91-4.09 |
| PFR/EDV | 0.67 | 0.234 | 0.35-1.29 |
| **Total Arrhythmia** | | | |
| Model 1: Classic Predictors (area under ROC curve = 0.68) | | | |
|  | OR | p-value | 95% CI |
| QRS Duration | 1.02 | 0.003 | 1.01-1.03 |
| RVEDVI (per 10 ml/m^2^) | 1.16 | 0.467 | 0.78-1.74 |
| RVESVI (per 10 ml/m^2^) | 0.80 | 0.535 | 0.40-1.62 |
| RVEF (%) | 0.96 | 0.362 | 0.88-1.05 |
| Model 2: Including Diastolic Predictors (area under ROC curve = 0.78) | | | |
| Age (years) | 1.06 | 0.003 | 1.02-1.10 |
| QRS duration | 1.02 | 0.024 | 1.00-1.03 |
| Indexed LA_min_ Vol. (per 10 ml/m^2^) | 1.70 | 0.110 | 0.89-3.25 |
| tPFR | 1.01 | 0.665 | 0.99-1.01 |
