# Supplementary material for: Association of cardiovascular magnetic resonance diastolic indices with arrhythmia in repaired Tetralogy of Fallot
Source: J Cardiovasc Magn Reson. 2023 Mar 13;25:17. doi: 10.1186/s12968-023-00928-x (PMC10009941; doi:10.1186/s12968-023-00928-x)
Supplement: Supplementary file 4 — Additional file 4: Table S4. Time to event analysis for arrhythmia. [file 12968_2023_928_MOESM4_ESM.docx]

**Additional file 4: Table S4.** Time to event analysis for arrhythmia

|  | Hazard Ratio AA (95% CI) | p-value | Hazard Ratio VA (95% CI) | p-value | Hazard Ratio TA (95% CI) | p-value |
| --- | --- | --- | --- | --- | --- | --- |
| **CMR Conventional Ventricular Parameters** | | | | | | |
| LVEDVI (ml/m^2^) | 1.00 (0.984-1.02) | 0.944 | **1.014 (1.003-1.025)** | **0.013** | 1.010 (0.994-1.017) | 0.333 |
| LVESVI (ml/m^2^) | 0.998 (0.976-1.021) | 0.893 | **1.017 (1.002 -1.032)** | **0.022** | 1.007 (0.993 -1.022) | 0.337 |
| LVEF (%) | 1.55 (0.035-67.861) | 0.820 | 0.025 (0.001-1.050) | 0.053 | 0.307 (0.016-6.045) | 0.438 |
| RVEDVI (ml/m^2^) | 1.002 (0.990-1.011) | 0.959 | 1.002 (0.992-1.012) | 0.691 | 0.996 (0.988 -1.010) | 0.420 |
| RVESVI (ml/m^2^) | 1.001 (0.987-1.014) | 0.937 | 1.006 (0.994-1.019) | 0.318 | 1.000 (0.987-1.010) | 0.800 |
| RVEF (%) | 4.221 (0.075-3236.413) | 0.483 | 0.052 (0.001-3.378) | 0.165 | 1.023 (0.039-26.682) | 0.989 |
| **LA Volume and Function** | | | | | | |
| Indexed LA_max_ Vol. (ml/m^2^) | 1.020 (0.981-1.061) | 0.321 | 1.038 (0.990-1.089) | 0.121 | 1.017 (0.982 -1.054) | 0.347 |
| Indexed LA_min_ Vol. (ml/m^2^) | 1.005 (0.953-1.060) | 0.855 | 1.031 (0.974-1.092) | 0.292 | 1.002 (0.955-1.051) | 0.937 |
| Indexed LA BAC Vol. (ml/m^2^) | 1.013 (0.973-1.053) | 0.528 | 1.021 (0.975 -1.069) | 0.371 | 1.005 (0.970-1.042) | 0.779 |
| Total LA Fxn (%) | 9.801 (0.141-680.661) | 0.291 | 0.634 (0.005-81.258) | 0.854 | 6.936 (0.176-273.390) | 0.302 |
| Passive LA Fxn (%) | 1.441 (0.030-69.927) | 0.277 | 4.917 (0.040-611.059) | 0.517 | 4.848 (0.151-155.206) | 0.372 |
| Active LA Fxn (%) | 78.905 (0.611-10193.89) | 0.078 | 3.353 (0.021-543.221) | 0.641 | 26.050 (0.455-1491.19) | 0.114 |
| **LV Diastology** | | | | | | |
| PER (ml/s) | **1.005 (1.000-1.010)** | **0.032** | **1.005 (1.001 -1.010)** | **0.022** | 1.003 (0.999-1.007) | 0.133 |
| tPER (ms) | 1.007 (0.997-1.017) | 0.168 | **1.021 (1.009 -1.033)** | **0.001** | **1.012 (1.003 -1.020)** | **0.007** |
| PER/EDV (s^-1^) | 0.951 (0.600-1.508) | 0.831 | 0.579 (0.323-1.036) | 0.066 | 0.807 (0.540-1.206) | 0.295 |
| PFR (ml/s) | 1.000 (0.996-1.003) | 0.871 | 1.002 (1.000-1.006) | 0.173 | 1.000 (0.998-1.004) | 0.760 |
| tPFR (ms) | 1.001 (0.995-1.006) | 0.240 | 0.998 (0.991-1.005) | 0.596 | 0.998 (0.992-1.003) | 0.461 |
| PFR/EDV (s^-1^) | 0.649 (0.390-1.081) | 0.097 | 0.569 (0.317-1.019) | 0.058 | 0.714 (0.468-1.089) | 0.117 |
